# The Association between “Knee Movement” Method and Traditional Radiograph Positioning Procedure with the Incidence of True Lateral Knee Radiograph Achieved

**DOI:** 10.5704/MOJ.2403.017

**Published:** 2024-03

**Authors:** CPB John, S Wendell, L Kevin, TS Earlene, AR Dio

**Affiliations:** 1 Department of Orthopaedics and Traumatology, Universitas Pelita Harapan, Tangerang, Indonesia; 2 Department of Orthopaedics and Traumatology, Universitas Pelita Harapan, Jakarta, Indonesia

**Keywords:** flexion-extension axis, knee osteoarthritis, knee radiographs, lateral knee radiographs, superimposition femoral condyle

## Abstract

**Introduction:**

This study aimed to find the association between the Knee Movement or KM method versus the traditional lateral knee radiograph positioning procedure and the incidence of true lateral knee radiographs achieved.

**Materials and methods:**

A cross-sectional study of patients with knee problems that underwent lateral knee radiograph using the knee movement method (KM method), starting from March 2022 until August 2022. Fifty knee radiograph results using the KM method (KM group) were compared to retrospective data from fifty knee radiograph from the patients before March 2022 using the traditional method of lateral knee radiograph as the control (TM group). The data were analysed using the Chi-Square test to see if the KM method is associated with more true lateral knee radiograph results achieved compared to the traditional procedure.

**Results:**

Fifty patients in the KM method group had 80% (n=40) true lateral knee radiographs and 20% (n=10) untrue lateral knee radiographs, while in the Traditional Procedure group from the retrospective data of 50 patients had 44% (n=22) true lateral knee radiographs and 56% (n=28) untrue lateral knee radiographs (P<0.05). There is no significant association between the type of procedure applied with the types of error (P=0.432). Nevertheless, it helps us as it gives a gross picture that most of the errors are under-rotation of the knee, either from the KM method Group 90% (n=9) or the Traditional procedure Group 79% (n=22).

**Conclusion:**

The KM method was associated with achievement of a more true and accurate lateral knee radiograph. Additional studies with a larger sample should be done to evaluate the reliability of this method.

## Introduction

Knee radiography remains a common and preferred examination for knee joint-related conditions. Radiography images are acquired mainly by acquiring both anteroposterior and lateral views. The true lateral view is essential for the diagnosis and pre-operative assessment of many knee-related conditions^1-3^. For patellar instability diagnostic purposes, the crossing sign and double contour sign are two of some objective evaluations of trochlear dysplasia that can only be seen clearly with the help of the true lateral position of the knee radiograph^2,4^. For purposes of pre-operative assessment in knee arthroplasty, the true lateral knee radiograph in 30 to 60° of flexion may be useful for measuring and evaluating the knee structure during the pre-operative assessment to help the surgeon predict the implant size and post-operative evaluation^5-8^.

However, in our experience, obtaining true lateral knee radiographs consistently is not easy in daily practice. The common positioning methods for the lateral knee radiograph in non-traumatic cases are a turned/rolled position (decubitus/non-weight-bearing) and a standing position (weight-bearing)^9^. While both methods are reliable, sometimes we found it difficult for our radiograph technician to adjust the patient’s limb to make the femoral condyle superimposed, both in supine and standing position. A study suggests, that to consistently achieve the true lateral view of the knee, the use of a fluoroscopy machine may be necessary^10^.

However, this is impractical due to equipment unavailability and increased radiation exposure. Due to these concerns, The Knee Movement method (KM method) is based on a previous concept by Dror Paley for assessing lower limb deformities with the use of knee movement as the reference to optimise the patient’s limb position without other modalities^11^. The KM method helps the radiograph technician to find the sagittal/flexion plane of the knee. Based on Dror Paley’s concept, it is easier to achieve the true lateral knee radiograph if the sagittal plane of the knee is parallel to the image receptor (IR)^11^. This study aimed to find the association between the KM method versus the traditional lateral knee radiograph positioning procedure and the incidence of true lateral knee radiographs achieved.

## Materials and Methods

Fifty knees of patients with knee problems underwent lateral radiographs using the knee movement method (KM group), starting from March 2022 until August 2022. compared to retrospective data from Fifty knee radiographs of the patients before March 2022 using the traditional method (TM group) of lateral knee radiograph. All patients included in this study were through a random sampling method. The exclusion criteria for the intervention group were (1) fracture at the femur and/or the tibia, (2) prior surgery on the hip and/or knees, (3) inability to move the hip or knee joint on the affected side freely, (4) inability to lie in lateral decubitus position comfortably. Consent statements were obtained from all participants of the KM group to receive the intervention. Ethical approval for this research was obtained from the Ethics Committee of Pelita Harapan University Faculty of Medicine, reference No. 160/K-LKJ/ETIK/VI/2023.

**Fig 1:**
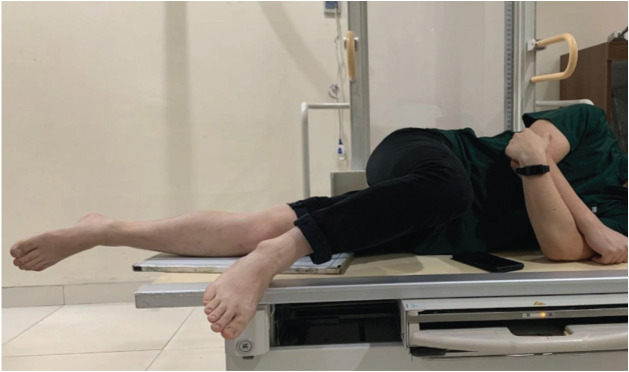
Lateral decubitus positioning. The contralateral knee is brought forward from the targeted knee.

**Fig 2:**
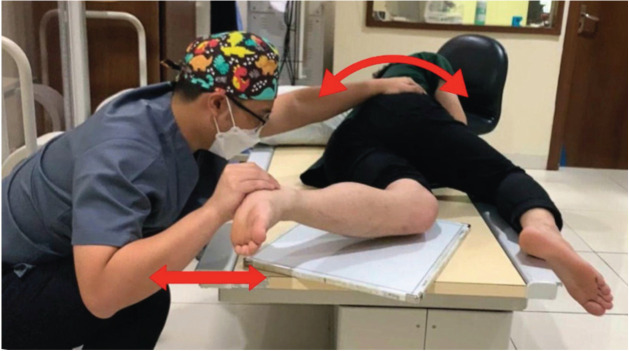
Knee bending and torso rotation. Passive bending and straightening of the knee between 30° to 90° while simultaneously rotating the torso backward or forward.

**Fig 3:**
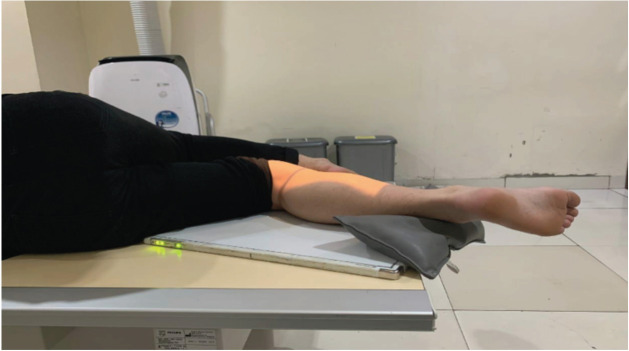
Padding on flexion plane to align the affected knee.

**Fig 4:**
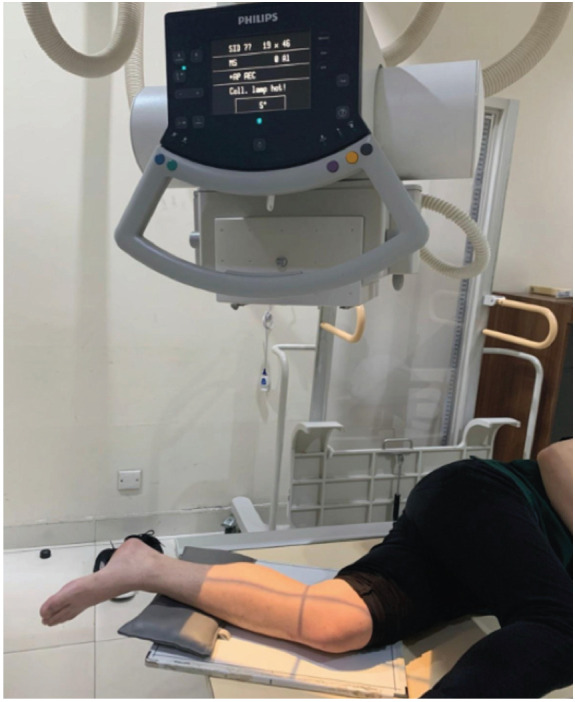
Padding on flexion plane to align the affected knee.

All lateral knee radiographs were shot using Philips DigitalDiagnost Rel 4.3. The kilovoltage peak is 60 kV with 5 milliamperes (mAs). The film-focus distance is 90cm. The KM method procedure is (1) the patient lies in a lateral decubitus position on the radiograph table (Fig. 1). (2) The contralateral knee is brought forward in front of the targeted knee. (3) The flexion plane is determined by passively bending and straightening the knee between 30° to 90° and simultaneously rotating the patient torso backward or forward until the flexion plane is parallel to the image receptor (Fig. 2). (4) The affected foot must be placed off the radiograph table to avoid internal rotation of the knee. If the patient has a large thigh, a pad should be placed under the ankle of the targeted leg, so the shin aligns with the thigh (Fig. 3). (5) The radiograph beam is directed 5° to 7° cephalad and the vertical beam is centred over the medial joint line of the knee. (6) The radiograph is then taken with the knee flexed over 30° to avoid rotation due to the tibia screw-home mechanism (Fig. 4).

The 20% rate of error was made while in the learning curve. We found several obstacles when performing this method.

The traditional lateral knee radiograph positioning procedure (Traditional Procedure) is (1) the patient stands in front and perpendicular to the image receptor. The contralateral knee is brought behind the targeted knee. (2) The targeted knee will be flexed to 30° and the foot will be placed parallel to the image receptor. (3) After arranging the patient's position, the radiograph beam is directed 5° to 7° cephalad, and the vertical beam is centred over the medial joint line of the knee.

To be defined as the true lateral view of the knee, it must meet these requirements: (1) Sufficient superimposition of the posterior aspect of the medial and lateral condyle. (2) The patella is clearly shown with clear patellofemoral joint space. (3) Minimal overlap of the tibial and femoral platform^9,12,13^. The knee radiograph results from the KM method procedure and traditional lateral knee radiograph positioning procedure will be categorised as true lateral knee radiographs and untrue lateral knee radiographs. Furthermore, the untrue lateral knee radiographs category will be divided into internal rotation error and external rotation error. The radiographs were assessed by the authors and reassessed by a senior radiologist using the INFINITT PACS 7.0 program for Windows. Before commencing the study, each assessor was provided with summary documents of methods outlined to assess each radiological measurement and instructed to review each radiograph independently from the other assessors and not to discuss or seek external help to determine a true lateral knee radiograph. All radiological measurements were recorded in a standardised and confidential database. All results were reviewed shortly after the radiograph was done.

The primary data were tabulated using Microsoft Excel 2016 and analysed using IBM SPSS Statistics for Windows version 25.0 to see the association between the variables. The association between the independent variables (KM method and traditional procedure) and the dependent variables (true lateral knee radiograph and untrue lateral knee radiograph) will be analysed using the Chi-Square test. In addition, the authors also analysed the association between the method used and the type of rotational error (internal rotation error vs. external rotation error) to find if there is any association.

## Results

**Fig 5:**
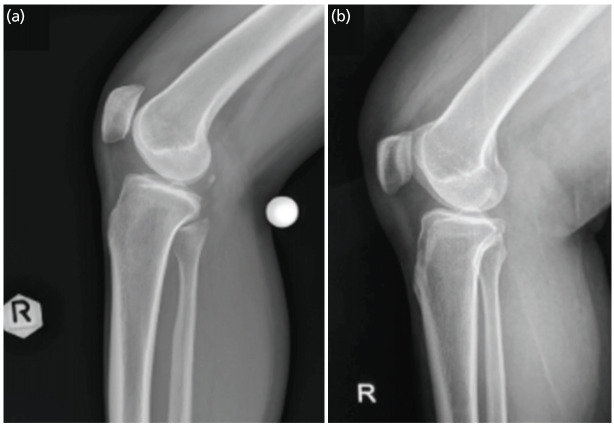
Direct comparison of the lateral knee imaging result from both methods. (a) An example results from the KM method. After the knee-flexion plane was adjusted to become parallel with the image receptor (IR), the femoral condyles were perfectly superimposed with a clear view of patellofemoral joint space, a true lateral knee radiograph. (b) Compared with an example result from the traditional procedure. Since the knee flexion plane was not parallel to the IR, the femoral condyles were not superimposed, and the patellofemoral joint space could not be seen.

At baseline, there were fifty patients with knee problems who underwent lateral knee radiographs with the KM method which were compared to the retrospective data from fifty knee radiographs from the patients before March 2022 using the traditional method (TM group). To be eligible for the intervention, patients with any of the following were excluded such as fracture at the femur and/or the tibia, any prior surgery on the hip and/or knees, the inability to move the hip or knee joint on the affected side freely, and inability to comfortably lie in lateral decubitus position. The outcome from the usage of two different methods was analysed with Chi-Square and shown in Table I which showed a correlation between two different methods where fifty patients in the KM method group had 80% (n=40) of true lateral knee radiographs and 20% (n=10) untrue lateral knee radiographs, while on the Traditional Procedure group from the retrospective data of fifty patients has 44% (n=22) true lateral knee radiographs and 56% (n=28) untrue lateral knee radiographs (P<0.05). Results also come with the data of types of errors in the untrue lateral knee radiographs results which are shown in Table II. There is no significant association between the type of procedure applied with the types of error (P=0.432). Nevertheless, it helps us as it gives a gross picture that most of the errors are under-rotation of the knee, either from the KM Method group 90% (n=90) or the Traditional Procedure group 79% (n=22). The example results of the lateral knee radiographs using the KM method (Fig. 5a) and the traditional procedure (Fig. 5b) were shown.

**Table I:** The association between types of radiograph procedures and the lateral knee radiographs outcomes.

Radiograph Procedure	n (%)	Results of Lateral Knee Radiographs		P-Value
		True n (%)	Untrue n (%)	
KM Method	50 (50)	40 (80)	10 (20)	.000
Traditional Procedure	50 (50)	22 (44)	28 (56)	
Total	34 (100)	62	38	

*p<0.05 indicates significant statistical results.

**Table II:** The association between types of radiograph procedures and the types of error in the untrue lateral knee radiographs.

Radiograph Procedure	n (%)	Results of Lateral Knee Radiographs		P-Value
		True n (%)	Untrue n (%)	
KM Method	10 (26)	9 (90)	1 (10)	.423
Traditional Procedure	28 (74)	22 (79)	6 (21)	
Total	38 (100)	31	7	

*p<0.05 indicates significant statistical results.

## Discussion

Obtaining true lateral knee radiograph is important for clinical practice. However, this topic is underreported. Based on our literature review, we did not find any studies that discuss the optimisation of the lateral knee radiograph without any modalities. We also did not find any studies that reported the disadvantages and difficulties of the current traditional method to obtain true lateral knee radiograph. A study by Wang *et al* tried to optimise the lateral position of the knee joint using CT images and MIP (maximum intensity projection) technique to obtain a true lateral radiograph^12^. Using the CT images and MIP technique has its advantages due to the image can be rotated or moved at any position or angle. However, the radiation dose and the operating cost are higher. Alternatively, the flexion-extension axis of the knee can be determined by palpating the adductor tubercle of the medial femoral condyle and the notch of the lateral femoral condyle. While it is possible, it needs special training for the radiology technician and sometimes it is difficult to do, especially in overweight patients concerning fat mass around the knee. The other way to determine the flexion-extension axis/flexion plane of the knee was used in this paper, by moving the knee and observing the flexion-extension motion orientation to determine the flexion plane. The concept was mentioned in 2002 by Dror Paley in Chapter 3 of Radiographic Assessment of Lower Limb Deformities^11^. They use this technique especially for patients with limb deformities to obtain a true long limb lateral view. To produce true lateral knee radiographs, they made the flexion plane of the knee parallel/in line with the image receptor. In this study, the flexion plane was determined by passively bending and straightening the knee between 30° to 90° while simultaneously rotating the patient's torso backward or forward until the flexion plane is parallel to the image receptor.

The results from both groups showed different outcomes, as an example shown in Fig. 5. This result hypothetically showed the possible circumstances in using the standing traditional procedure alone causing the knee-flexion axis to lie unparallel to the image receptor which leads to the absence of superimposition from both condyles achieved. In this study, the KM method encourages the technician and assessors to assess the knee-flexion axis to obtain axes that were parallel to the image receptor before achieving the lateral knee images.

In our experience, this method is easier to learn for the radiology technician. The method can be reliable because of the variability of knee orientation in humans. The orientation of the knee may correlate with the foot progression angle (FPA). FPA is defined as the angle made by the long axis of the foot from the 2nd metatarsal to the calcaneus and the line of progression of gait^14^. The normal average FPA ranges from 5° in children to 13° in adults^15,16^. The FPA can be largely influenced by femoral neck orientation (eg. hip anteversion and retroversion)^17^. The orientation of the femoral neck may also influence the orientation of the knee and its flexion plane. However, the FPA can also be influenced by tibial torsion by looking at the thigh-foot angle (TFA). The TFA is the angle between the axis of the foot and the axis of the thigh. The degree of tibial torsion is determined by measuring it in a prone position while the knee is flexed at 90°.

Four knee radiographs have under-rotation errors due to the foot interference on the table making the internal rotation of the knee. Five knee radiographs were taken from obese patients with all of the radiograph results being under-rotation of the knee. The under-rotation errors were also suspected due to the home-screw mechanism occurrence because of inadequate flexion. From nine knee radiographs with under-rotation error, four knee radiographs were taken with less than 30° of flexion. To solve these problems, the decision was made to set the foot off the table. A pad was added under the ankle in obese patients to make the shin align with the thigh. Unfortunately, we did not measure the BMI or the knee diameter of our patients, which can be useful parameters to predict the risk of under-rotation error. The knee was set roughly to more flexion at 45° to 80°, as a safe zone to avoid the screw-home mechanism. The last issue came when the patient’s torso must be set in an extreme position and some patients were unable to withhold the position, for example, the patients with back pain, hip pain, or general weakness. Thus, the patient will be excluded from the study if they cannot maintain the extreme torso position. In the group that received the TM method, 22 knees were dealing with under-rotation errors. The suspected cause is the positioning of the knee based on foot position, where the foot must be parallel to the image receptor. This can lead to an unnatural position of the knee in some patients because of the variability of the FPA. Since our method is heavily based on knee movements, patients with grade IV knee osteoarthritis might not participate in our study. However, after we try to include patients with grade IV osteoarthritis, it seems like it depends on the individual pain tolerance level. Some patients with painful grade IV osteoarthritis were excluded from the study. However, we still include some patients with grade IV osteoarthritis with good pain tolerance.

The major limitation of the study is using standing vs. decubitus position as a direct comparison. However, while the standing position might cause joint space height difference of the femorotibial joint compared to the decubitus position^18^, the main focus of lateral knee radiograph is not to evaluate the femorotibial communal space. Note that in the context of pre-operative assessment of total knee replacement for knee osteoarthritis, both anteroposterior (AP) and lateral views of the knee will be required, the AP views will be used to evaluate the joint space^19^. Our study also didn’t measure the timing to conduct the KM method, compared with the traditional procedure. Therefore, further investigations with detailed timing for both groups will be valuable in future studies. The KM method may also be applicable in standing by using the lunging position and driving the knee forward and backward while simultaneously externally and internally rotating the foot until the flexion plane is parallel/in line with the image receptor.

## Conclusion

The KM method was associated with achievement of more true lateral knee radiograph results. Additional studies with a larger sample should be done to evaluate the reliability of this method.
